# Case of Cataract in Both Eyes; Occurrence of the Affection in the Males of Three Generations

**Published:** 1846-12

**Authors:** Samuel Sumner Dyer

**Affiliations:** Late House Surgeon to King’s College Hospital


*Case of Cataract in both eyes ; occurrence of the affection in the males of three generations.* By Samuel Sumner Dyer, M. R. C. S. Late House Surgeon to King's College Hospital.—William Wiseman, aged 62, originally a labourer, but latterly, from his inability to work, through total blindness, living unemployed upon parochial allowance, a native of Burley, in the New Forest, where he has always resided, applied to me in February last, and made the following statement:—

That he was one of a large family; that his father had never had good sight from childhood, becoming totally blind at fifty-four years of age, but was afterwards operated upon for cataract, and was much benefitted by the operation, being up to the time of his death, which happened some years after, enabled to read small print with facility; that the sight of his sisters was good—that of the brothers always imperfect; one died young—another now resident near this town is quite blind. He, himself, "always had a mist before his eyes;" has been blind for twenty-one years, and unable to distinguish dark from light for the last two years. He has two sons, both with imperfect vision, (in one a cataract is forming,) and a daughter, whose eyes are perfect. The second son is married and has a large family, the sons are purblind, the sight of the daughters being perfect. I have seen all these individuals at different times. Their eyes have a peculiar lustre, the pupils act well, but there is that constant motion of the eyeball, evidently dependent on its not being fixed on any particular object.

The man, who applied to me, remembering his father's case, was very anxious that something should be done in his, and as his general health was excellent, and he could distinguish a bright light when the pupils were acted upon by belladona, I felt but little hesitation in complying with his request. Accordingly, I performed the operation of depression in both eyes during the last month, my father fixing the eyes with Pellier's speculum, whilst I used the needle recommended by Scarpa. The steps of the operation were exactly those generally laid down for our guidance, therefore I need not trespass too much on your Journal or your reader's time by enumerating them. The eyes have been kept covered with a light piece of linen up to the present time,—eighteen days from the operation,—when he can distinguish any object (even of the size of a shilling,) at seven yards distance. Very little inflammation has resulted from the operation, and the only after-treatment has consisted in partial exclusion of light, the application of cold water, and the administration of three doses of a purgative medicine.

The points which I consider of so great interest in this case are—1st, the transmission of the disease to only the male subjects of three generations, none of these escaping, whilst all the females do; 2nd, the termination of the imperfect vision at a certain period, in cataract; 3rd, the improvement in sight after that was removed, even compared with what it was before its fo mation; 4th, the fact of the nerve of sight retaining its power, though uncalled for, for so long a time; and lastly the slight inflammation succeeding the above operation.—*Ibid.*

